# Dr. Hamilton’s Lecture on Phrenology

**Published:** 1845-06-01

**Authors:** 


Dr. Hamilton's Lecture on Phrenology.—Although Phrenology addresses itself particularly to the consideration of Medical men, and, if true, possesses relations which are interesting and important in medical investigations, we do not propose to devote to it space which can be more appropriately occupied with subjects more directly relevant to practical medicine. On this account we must content ourselves with acknowledging the receipt of a copy of the above lecture, which our readers will find advertised to be obtained at our Publisher's office. The lecture is a candid exhibition of the arguments *against* the validity of the claims of Phrenology to be ranked as a true Science, and is worthy the attention of those who wish to look on both sides of the question.

